# 

**Published:** 1915-12

**Authors:** 


Contents, Page 5. Index to Advertisements, Page 6. Publicity Dept., Page 20.

Yearly Vol. 71 December 1915 Number 5

### Buffalo Medical Journal

Established 1845 by AUSTIN FLINT, M. D.

EDITOR AND PUBLISHER:
A. L. BENEDICT, A.M., M.D. 228 Summer Street, Buffalo, N. Y.

ASSOCIATE EDITORS:

New York State:

Grover W. Wende, M. D., Buffalo.

C. W. Hennington, B.S., M.D., Rochester

Charles Haase, M. D., Elmira.

Willis E. Ford, M. D., Utica.

Martin B. Tinker, B. S., M. D., Ithaca.

H. I. Davenport, A. M., M. D., Auburn.

Foreign

Sir William Osler, M. D., LL. D., Oxford, Eng.

Prof. P. K. Pel, M. D., Amsterdam, Holland.

Prof. C. A. Ewald, M. D., Berlin, Germany.

Rene Gaultier, M. D., Paris, France.

J. George Adami, M. D., LL.D., F. R. S. Montreal, Can.

Henry W. Hill, LL. D., Buffalo, Associate Editor in Medical Jurisprudence.

